# Consensus Guidelines for Digital Scholarship in Academic Promotion

**DOI:** 10.5811/westjem.2020.4.46441

**Published:** 2020-07-08

**Authors:** Abbas Husain, Zachary Repanshek, Manpreet Singh, Felix Ankel, Jennifer Beck-Esmay, Daniel Cabrera, Teresa M. Chan, Robert Cooney, Michael Gisondi, Michael Gottlieb, Jay Khadpe, Jennifer Repanshek, Jessica Mason, Dimitrios Papanagnou, Jeff Riddell, N. Seth Trueger, Fareen Zaver, Emily Brumfield

**Affiliations:** * Staten Island University Hospital - Northwell Health, Department of Emergency Medicine, Staten Island, New York; † Lewis Katz School of Medicine at Temple University, Department of Emergency Medicine, Philadelphia, Pennsylvania; ‡ University of California, Los Angeles Medical School, Department of Emergency Medicine, Los Angeles, California; § University of Minnesota Medical School, Department of Emergency Medicine, Minneapolis, Minnesota; ¶ Mount Sinai St. Luke’s-West, Department of Emergency Medicine, New York, New York; || Mayo Clinic College of Medicine, Department of Emergency Medicine, Rochester, Minnesota; # McMaster University, Department of Medicine, Hamilton, Ontario; ** Geisinger Medical Center, Department of Emergency Medicine, Danville, Pennsylvania; †† Stanford University School of Medicine, Department of Emergency Medicine, Stanford, California; ‡‡ Rush University Medical Center, Department of Emergency Medicine, Chicago, Illinois; §§ University of Florida College of Medicine, Department of Emergency Medicine, Jacksonville, Florida; ¶¶ Oschner Clinic Foundation, Department of Emergency Medicine, New Orleans, Louisiana; |||| University of San Francisco-Fresno, Department of Emergency Medicine, Fresno, California; ## Sidney Kimmel Medical College at Thomas Jefferson University, Department of Emergency Medicine, Philadelphia, Pennsylvania; *** University of Southern California Keck School of Medicine, Department of Emergency Medicine, Los Angeles, California; ††† Northwestern University, Department of Emergency Medicine, Chicago, Illinois; ‡‡‡ University of Calgary, Department of Emergency Medicine, Calgary, Alberta, Canada

## Abstract

**Introduction:**

As scholarship moves into the digital sphere, applicant and promotion and tenure (P&T) committee members lack formal guidance on evaluating the impact of digital scholarly work. The P&T process requires the appraisal of individual scholarly impact in comparison to scholars across institutions and disciplines. As dissemination methods evolve in the digital era, we must adapt traditional P&T processes to include emerging forms of digital scholarship.

**Methods:**

We conducted a blended, expert consensus procedure using a nominal group process to create a consensus document at the Council of Emergency Medicine Residency Directors Academic Assembly on April 1, 2019.

**Results:**

We discussed consensus guidelines for evaluation and promotion of digital scholarship with the intent to develop specific, evidence-supported recommendations to P&T committees and applicants. These recommendations included the following: demonstrate scholarship criteria; provide external evidence of impact; and include digital peer-review roles. As traditional scholarship continues to evolve within the digital realm, academic medicine should adapt how that scholarship is evaluated. P&T committees in academic medicine are at the epicenter for supporting this changing paradigm in scholarship.

**Conclusion:**

P&T committees can critically appraise the quality and impact of digital scholarship using specific, validated tools. Applicants for appointment and promotion should highlight and prepare their digital scholarship to specifically address quality, impact, breadth, and relevance. It is our goal to provide specific, timely guidance for both stakeholders to recognize the value of digital scholarship in advancing our field.

## INTRODUCTION

The promotion and tenure (P&T) process requires the appraisal of individual scholarly impact in comparison to scholars across institutions and disciplines. Comparative metrics such as the journal impact factor and the h-index are used to quantify and compare the quality of an individual’s scholarship and, therefore, his or her academic merit.^1^ As knowledge dissemination methods evolve in the digital era, we must adapt traditional P&T processes to include emerging forms of digital scholarship.^2^ In this paper, we aim to first situate our readers within the literature on the topic of academic scholarship, after which we will describe the process by which we derived and refined our consensus guideline. Finally, we will outline the recommendations for the use of digital scholarship for academic promotion made by this particular guideline group.

### The Evolution of Scholarship

Scholarship is persistently dynamic. Analog technologies progressed from tablet and stone to pen and paper; modern digital scholarship is evolving with blogs, podcasts, and digital journals. Still, the standards for evaluation are consistent and focus predominantly on impact and quality of the scholarship.^3^ In 1990, Ernest Boyer of the Carnegie Foundation originally redefined scholarship for the professoriate as belonging to one of four types.^4^ A decade later, Charles Glassick followed up this work by describing criteria for evaluating scholarship.^5^,^6^ To further develop nuances around the scholarship of teaching and learning, Lee Shulman and Patricia Hutchings further clarified specific criteria for this subtype of scholarship (to differentiate it from high-quality, scholarly, and evidence-based teaching).^7^ These foundational concepts are summarized in Table 1 below.

Traditionally, peer-review processes of academic journals served as a safeguard to ensure overall quality, with evaluators deferring to experts and peers within a scholar’s domain to provide an appraisal for quality and an estimate of impact. Similarly, bibliometrics of journals (eg, journal impact factor)^8^ and number of citations are surrogates for scholarly reach and proof of impact.^3^ Despite well-described limitations, these metrics are quantifiable and defined processes that are easily compared. Thus, they are highly relied upon by P&T committees to compare scholars from disparate disciplines. When scholarship using new media is produced, it is reasonable to scrutinize the methodology, content, impact, and quality of these new forms of scholarship, such as digital scholarship. Our use of the term “digital scholarship” in this paper reflects original content that is disseminated digitally, whether that content is research, teaching materials, enduring resources, commentaries, or other scholarly work.

It is unsurprising that as the world becomes more digital, so do scholarly contributions.^9^ Online-only journals, pre-print archives,^10^ and post-production, peer-review journals (eg, *Cureus*) are rapidly changing the landscape of peer-reviewed publication.^11^,^12^ Similarly, with the advent of peer-reviewed blogs,^13^ self-published peer-reviewed books,^14^ and educational resource repositories, we see an increased breadth of expression from those engaging in the scholarship of teaching. These varied forms increasingly mirror the rigor required by Glassick’s criteria and Shulman’s paradigms.^15^,^16^

### Quantity vs Quality

Judging these new forms of scholarship is different. In many ways, with advanced web analytics, it is easier to quantify the reach and attention (eg, pageviews, podcast downloads, IP addresses that have accessed the content, and time on page) of these digital assets. (See Table 2 for common analytics available for new digital scholarship.) For example, the PubMed-indexed repository MedEdPortal provides download analytics of the published resources that aid in describing entries as fitting within the scholarship of teaching.

However, since many disciplines both within and outside of medicine have not yet fully embraced digital scholarship as enthusiastically as emergency medicine (EM) and critical care,^17^ it is no surprise that P&T committees do not yet have specific or universal standards for presentation or evaluation of digital scholarship.^18^ Those without digital scholarship experience may grapple with understanding the nuances of determining impact and quality in this new era, and their lack of understanding may even result in general skepticism of novel products. Thus, fields that have already established robust methods for determining the quality of digital scholarship can lead the way. Since digital scholarship has matured in EM,^19^,^20^ it is appropriate for our field to call for the identification of best practices for evaluating digital scholarship and for consensus in the inclusion of such items in promotions decisions.

Specific guidelines for P&T are lacking despite robust digital contributions proliferating among academicians. In this work, we provide a guiding framework for the presentation and evaluation of digital scholarship for the applicant for promotion, referees for the candidate, and members of P&T committees.

## METHODS

We conducted a blended, expert consensus procedure using a nominal group process to create a consensus document.^21^ Invited participants met at the Council of Emergency Medicine Residency Directors (CORD) Academic Assembly on April 1, 2019 (Seattle, WA), to discuss recommendations for evaluation and promotion of digital scholarship with the intent to develop specific, evidence-supported recommendations to P&T committees and applicants. We began with a live, brainstorming event. The meeting notes were compiled by a leadership team and formatted into a collaborative working document. All authors continued formulating this document via a collaborative online authorship using Google Docs (Google LLC, Mountain View, CA).^22^

### Participants

The participants were selected by the leadership of the CORD Social Media and Digital Scholarship Committee (EB, ZR, AH). Participants were selected based on criteria of known interest or scholarship in the area, national and international level contributions to EM digital scholarship, and availability to attend the conference in person or by phone. Supplemental Digital Appendix A lists original invitation list and individual selection rationale. The complete list of attendees of the in-conference proceedings is listed in the acknowledgments.

### Procedures

As a large group, the consensus conference participants democratically developed the discussion and brainstorming procedures. Based on suggestions from the floor about previous consensus procedures at other similar conferences,^23^,^24^ our group decided to engage in small- group brainstorming discussions aligned with the expertise and interests of the participants, which was then discussed as a large group and vetted by the rest of the participants. Consensus was defined as universal agreement of the participants.

### Ideation and refinement

The participants self-identified their areas of expertise or interest, and then separated into three groups based on these content areas using an iterative process to formulate specific recommendations. The three discussion groups were tasked with formulating recommendations for the following:

1. The P&T applicant for promotion of one’s digital scholarship;
2. P&T committee members for evaluation of quality of digital scholarship;
3. P&T committee members for evaluation of the impact of digital scholarship.

Small groups presented preliminary recommendations to the entire group and made further revisions via iterative discussion. Participants transcribed an outline of the discussion and final recommendations and agreed upon them in a democratic fashion. Participants self-selected areas of the manuscript to prepare based on expertise, interest and group approval. All members developed the manuscript from the outline via collaborative authorship. All participants contributed to the manuscript, and CORD Social Media and Digital Scholarship committee members (AH, MS, ZR, EB) served as final editors of the manuscript.

## RESULTS

### Recommendations for Presenting Digital Scholarship for Promotion and Tenure

#### Demonstrate Scholarship Criteria

When presenting digital scholarship to a P&T committee, begin by ensuring and demonstrating that it meets the criteria of scholarship as defined by Glassick and expanded upon by Sherbino and colleagues with regard to social media.^25^,^26^ The adapted criteria are as follows: 1) create original content; 2) advance the field of health professions education by building on theory, research or best practice; 3) be archived and disseminated, and 4) provide the health professions education community with the ability to comment on and provide feedback in a transparent fashion that informs wider discussion. In addition, consider providing evidence of archival and dissemination, such as Google Scholar indexing or inclusion of a digital object identifier (DOI).

#### Provide External Evidence of Impact

Ensure that your digital scholarship is reflected consistently throughout your promotions dossier. Dissemination metrics are important to include as measures of impact. For example, some blog editors will provide information about how many times a post has been accessed and the locations of its readers, if requested for P&T purposes. Such metrics of dissemination and impact should be presented in the dossier as evidence of your professional reputation as a scholar in your field.

Additional metrics include pageviews, downloads, and geographic reach. Other programs assessing the reach of scholarship, such as altmetrics, may also be valuable.^27^ The Social Media Index is a relatively newer technique to assess the impact of websites and could be used as a surrogate for impact, much the same as a journal’s impact factor.^28^ See Table 2.

Other measures of impact could include letters of support and awards. If permitted by your institution, consider obtaining letters of support with regard to your digital scholarship. You may also consider inviting both peer letters and letters from non-collaborators discussing the dissemination metrics and impact of specific pieces of scholarship, or simply your overall impact. There are also a number of digital scholarship-based awards, which may be of value for demonstrating scholarly impact.^29^

#### Include Digital Peer-review Roles

Include editor or peer-reviewer roles for digital scholarly content in your curriculum vitae (CV) in a similar manner as you would for traditional print literature. It is important to highlight these supporting components of digital scholarship and they should be factored into the P&T decisions.^25^

#### Citing Digital Scholarship

Cite scholarly work on your CV using a consistent format, whether that work was published in a hard-copy journal or as digital content. Reorganize the categories of scholarly publications on your CV to include a section for “Digital Scholarship,” which is the appropriate subheading for items such as blog posts, podcasts, and videos. See Table 3 below for example subheadings for the scholarly bibliography of your CV. Include only those items that reflect true scholarship and relate to the health professions or sciences. Do not list citations for personal website posts or other digital content that is unrelated to your academic position.

Consistently format your scholarship across all subheadings on your CV following the *American Medical Association (AMA) Manual of Style, 10th Ed*.^30^ The *AMA Manual* describes the methods for citing scholarship in most of the categories listed. Examples of each citation type are provided above, and selected citations are adapted in Table 4. Digital scholarship is best formatted using the *AMA Manual* instructions for “Internet Documents.”^33^ *Academic Life in Emergency Medicine* (ALiEM) also offers guidelines for citing digital scholarship, with examples.^31^

Digital scholarship is often criticized for lack of peer review, which leads to confusion about the quality and integrity of articles published in exclusively online journals. Peer review is a requirement for all journals to be indexed and available on PubMed, including online journals. Research articles published in online-only journals that have a PubMed unique identifier (PMID)^32^ should *not* be listed under “Digital Scholarship,” but rather alongside similar scholarly work published in peer-reviewed print journals. Regardless of the mode of publication, all peer-reviewed research should be listed under the same CV subheading in the “highest” possible category.

Blog posts that are cited under a “Digital Scholarship” CV subheading can be peer reviewed as well. For example, some blogs offer a peer-review process for authors and identify which posts have undergone peer review.^33^ Therefore, use qualifiers to identify any digital scholarship citation on your CV that was peer-reviewed or invited. These qualifiers may add additional credibility to your scholarship when a P&T committee reviews your CV.

## DISCUSSION

### Crafting a Digital Scholarship Mission Statement

A digital scholarship mission statement can provide a framework for your P&T committee to understand and interpret your digital scholarship.^34^ Akin to the educational philosophy statement of a teaching portfolio, the digital scholarship mission statement provides a lens through which the committee can interpret the congruence and value of your scholarship.^35^,^36^ This narrative should articulate the beliefs that drive your digital work in ways that give perspective to your activities and provide consistency with the academic and social media strategies of each institution. Table 5 below lists specific considerations to include. Please see Supplemental Digital Appendix B for a sample narrative.

### Use Traditional Frameworks: Harnessing the Teaching Portfolio

We recommend using traditional frameworks to describe digital scholarly activity and support for academic promotion. One such example of this is the teaching portfolio. As not all institutions require a separate educational portfolio, we recommend that you present your digital scholarship alongside traditional scholarship according to your institutional requirements. Refer to your respective institutional guidelines for requirements and formatting of teaching portfolios. Regardless, to facilitate appraisal by P&T committees you should create a dossier that includes a digital mission statement, demonstrates alignment with overall career development goals, and describes the scholarly significance of your digital work.^25^

Digital scholarship should not replace materials that are typically included in a teaching portfolio, such as course evaluations or other traditional measures of teaching effectiveness. Teaching portfolios should summarize teaching *effort* and *quality* that meet the criteria of Boyer’s scholarship of teaching.^4^,^37^ Within the teaching portfolio, you may reflect and provide exemplars of digital works and curricula that you have created or curated for learners, but you will not actually list item-by-item the digital scholarship you produce; this should take place in the CV. An entry in a portfolio would holistically describe the pedagogical principles behind a digital educational program or innovation (eg, if you are the creator of a popular podcast, you would explain how you developed the podcast, how you engaged stakeholders to develop the podcast, and, if possible, share data to convey its impact at large through analytics). In contrast, entries of digital scholarship on a CV would be entered individually. Table 6 provides some common examples of digital scholarship, and how they might align best with previous descriptions of traditional academic scholarship (as per Boyer, Glassick, Hutchings and Shulman).

### Appraising Impact

There are no hard and fast rules for determining impact. Cabrera and his colleagues have previously suggested scale-based assessments of social media-based impact in their 2017 paper.^34^ They provide ample guidance to promotions committees for comparing size and scale of various media within a specific subtype (eg, international blog vs a local blog). We highly recommend that readers review this article for further guidance.

Another tool is the Social Media Index, which seeks to create an “impact factor”-like metric based on social media followership. This tool would be best used to judge the impact of an entire digital media collection, such as an entire website or podcast. This tool is available online ( https://www.aliem.com/social-media-index/) and has been revised and validated against quality metrics within emergency medicine Free Open Access Medicine resources.^38^

### Appraising Quality

Due to lower barriers of entry allowing digital scholarship to be more easily produced, general skepticism due to less serious, nonmedical online content, as well as pseudoscientific and/or predatory online content, groups have sought to scaffold and support end-users and educators in seeking high-quality online resources.^39^,^40^ The online medical education community has worked to quell skepticism by establishing methods to appraise the quality of digital scholarship.^3^ See below for a list of critical appraisal tools for rating online secondary resources. For those who have been asked to review files as external referees, these tools may be very useful in guiding us toward high-quality educational content from an educator’s CV or portfolio.

Some scholars in this space have proposed that we move beyond bibliometrics and surrogates for quality (eg, impact factor, citations, altmetrics), and that P&T committees consider applying direct quality assessments to items of interest (eg, applying the revised METRIQ^41^ or ALiEM Approved Instructional Resources (AIR series) scores^39^ to a few choice works of digital scholarship from a faculty member’s CV, or applying the PRISMA^42^ reporting guidelines to a few systematic reviews). Equitably applying both descriptive bibliometrics (eg, citation rate, h-index, etc.) and quality audits to all works of scholarship (digital or otherwise) would go a long way to augment P&T processes. Table 2 contains suggested critical appraisal tools to facilitate secondary resource evaluation.

## LIMITATIONS

The live conference was limited to invited participants who could join in person or by phone. Those with scheduling conflicts were therefore excluded from the live session, perhaps limiting valuable insights and contributions. However, those that could not attend the live conference were still heavily involved in the organization and creation of the recommendations post-conference via a collaborative writing process. Additionally, all authors participated robustly in the asynchronous editing of this manuscript, reducing the potential that important viewpoints were excluded. Conference participants were selected by the committee members, and important contributors may have been overlooked. To reduce this possibility, invited members were requested to suggest additional invitees. Finally, as digital scholarship participants and creators, there may be bias toward legitimizing our own work over less-familiar scholarship.

We attempted to ground our recommendations using best available evidence in order to reduce this bias. However, there is certainly a paucity of literature on how social media is viewed upon (or accepted) as a form of scholarship by the academy. Thus, further explorations of the acceptability or evaluation of digital by P&T committees may be a useful program of research going forward. A paper has recently been published about perceptions in the librarian sciences world that is quite interesting, and worthy of replication within academic medicine.^43^

## CONCLUSION

As traditional scholarship continues to evolve within the digital realm, academic medicine must also adapt how that scholarship is evaluated. P&T committees in academia are at the epicenter for supporting the changing paradigm in scholarship. Unlike traditional academic products, where reach and impact were difficult to quantify, web-based metrics allow us to track unique users and their locations. The authors suggest that committees critically appraise digital scholarship using the methods outlined in this paper. Applicants for appointment and promotion should highlight and prepare their digital scholarship in a way that specifically addresses quality, impact, breadth, and relevance. It is our goal to provide specific, timely guidance for both stakeholders to recognize the value of digital scholarship in advancing our field.

## Supplementary Information





## Figures and Tables

**Table 1:** Foundations of education and teaching scholarship.

Boyer’s Scholarly Domains 1990	Hutchings and Shulman Criteria for Scholarship of Teaching 1999	Glassick’s Criteria for Evaluating Scholarship 2000
Scholarship of discovery Scholarship of integration Scholarship of application Scholarship of teaching	Public Available for peer review & critique according to the standards of a field Able to be reproduced and extended by other scholars	Clear goals Adequate preparation Appropriate methods Significant results Effective presentation Reflective critique

**Table 2:** Summary of metrics used to demonstrate digital scholarship impact, role and quality, with a sample scholarly work.

Promotion metric	Supporting data	Example with metrics
Impact *Demonstration of impact shows your work reaches your intended audience*	Pageviews Time Spent on Page Likes Impressions Dissemination (Shares) Unique Users Geographic Reach Followers on Professional Social Media Accounts Social Media Index Digital Object Identifier (DOI) Alexa Ranking Altmetrics	Thoma B, Chan T, Benitez J, Lin M. Educational Scholarship in the Digital Age: A Scoping Review and Analysis of Scholarly Products. *The Winnower*. 2014. doi:10.15200/winn.141827.77297 Pageviews 4137 Altmetric Score 61 202 tweets from 86 users, with an upper bound of 263,362 followers
Role *Demonstration of your “brand” or role within digital scholarship helps establish your area of expertise*	Editor Author Curator Reviewer Invited Commentaries Podcast Guest or Editor	[Invited Commentary] Berg A, Weston V, Gisondi MA. Journal Club: Coronary CT Angiography Versus Traditional Care. NUEM Blog. http://www.nuemblog.com/blog/cta-for-chest-pain/ Published online 4/12/16.
Quality *While also demonstrating commitment to scientific rigor in your work, you may also highlight novel quality assurance methods unique to digital scholarship*.	METRIQ-5 and -8, rMETRIQ ALiEM AIR Score SAEM Online Academic Resources (SOAR) Social Media Index (SMi) The Quality Checklists for Health Professions Blogs and Podcasts	[Peer-reviewed blog] Long, B. “Myths in Heart Failure: Part I - ED Evaluation” emDOCs.net http://www.emdocs.net/myths-in-heart-failure-part-i-ed-evaluation/ published online 7/23/2018. Selected as ALIEM AIR Cardiovascular, Non-ACS module 2019.^39^ This post was deemed to be of an acceptable score within the ALiEM AIR Scoring tool, and was granted the designation “AIR Approved” by the adjudicating group of educators. There is a second tier below, known as “honorable mention” for posts of moderate quality that did not meet the threshold for inclusion.

*ALiEM*, Academic Life in Emergency Medicine; *AIR*, approved instructional resources; *SAEM*, Society for Academic Emergency Medicine

**Table 3:** Subheadings for “Scholarly Bibliography” section of curriculum vitae.

1	Original Research Articles - Peer Reviewed
2	Editorials, Reviews, Case Reports, Letters, Commentaries - Peer Reviewed
3	Textbooks, Textbook Chapters
4	Proceedings and Non-Refereed Papers
5	Digital Scholarship
6	Abstracts
7	Exhibits, Audiovisuals, Teaching Materials
8	Media Appearances and Quotes - Print, Television, Online

**Table 4:** Suggested examples of digital scholarship citations and qualifier use.

Format:
Last Name, First Initial. “Title of Submission.” Name of Publisher. URL as hyperlink. Published online XX/XX/XX.
Example:
Gisondi MA, Stefanac L. “The Feedback Formula: Part 1, Giving Feedback.” International Clinician Educators Blog. https://icenetblog.royalcollege.ca/2018/10/02/the-feedback-formula-part-1-giving-feedback/. Published online 10/02/18.
Example Qualifiers for Curriculum Vita:
[Blog Post] Gisondi MA. “Leadership in Medical Education: Addressing Sexual Harassment in Science and Medicine.”International Clinician Educators Blog. https://icenetblog.royalcollege.ca/2019/01/15/leadership-in-medical-education-addressing-sexual-harassment-in-science-and-medicine/ Published online 1/15/19.
[Podcast Guest] Kellogg A, Gisondi MA. “Sex and Why Episode 10: How to Give Better Feedback.” In: seX & whY Podcast (Wolfe J, Editor-in-Chief.) https://www.sexandwhy.com/sex-why-episode-10-how-to-give-better-feedback/ Published online 1/29/19.
[Peer-Reviewed] Schnapp B, Fant A, Powell E, Richards C, Gisondi M. “8 Tips for How-to-Run an Awesome Works-in-Progress Meeting.”Academic Life in Emergency Medicine. http://www.aliem.com/8-tips-works-progress-meeting/ Published online 11/1/15.
[Commentary, Invited] Berg A, Weston V, Gisondi MA. Journal Club: Coronary CT Angiography Versus Traditional Care. NUEM Blog. http://www.nuemblog.com/blog/cta-for-chest-pain/ Published online 4/12/16.
[Video] Mason J. Placing a Transvenous Pacemaker. Emergency Medicine: Reviews and Perspectives. October 1, 2018. https://www.emrap.org/episode/transvenous/transvenous. Accessed November 21, 2018.
[Traditional Paper with Altmetrics] Chan TM, Gottlieb M, Sherbino J, Cooney R, Boysen-Osborn M, Swaminathan A, Ankel F, Yarris LM. The ALiEM faculty incubator: a novel online approach to faculty development in education scholarship. Academic Medicine. 2018 Oct 1;93(10):1497–502. Altmetrics data: https://wolterskluwer.altmetric.com/details/43542602

**Table 5:** Specific elements to consider within a mission statement.

1	Reinforce why your digital scholarship exists and is important to the field.
2	Explain your digital scholarship’s broad goals and objectives.
3	Explain your perception of needs in the modern learning environment, and how that affects your methods.
4	Explain how your approach to digital scholarship/teaching has changed over time.
5	Explain the niche that you are filling, specifically highlighting how your role/expertise at your institution gives you a reputable voice.
6	Describe how your digital scholarship complements your other, more traditional forms of scholarship.
7	Explain how digital scholarship aligns with your overall career objectives.
8	Name your intended target audience and describe other collateral audience groups that may benefit from your public academic work.
9	Describe best practices for ensuring quality during the content creation process: Highlight team-based and interdisciplinary scholarship as markers of quality Preview external validation processes of your digital scholarship (below).
10	Highlight the ancillary benefits that have arisen because of your digital scholarship presence, such as invited lectures or collaborations on additional scholarship.

**Table 6:** How types of digital scholarship might be described using traditional descriptions of academic scholarship.

	Blogging	Podcasting	Tweeting
Example of digital scholarship	Blog post providing a new insight into a novel teaching technique, with a recipe for helping students learn about social justice by meeting patient partners.	Podcast synthesizing the role of human factor engineering in the emergency department.	Tweetorial reviewing and appraising the latest evidence on a topic
Does this meet the criteria for scholarship per Hutchings and Shulman? Public Available for peer review and critique according to the standards of a field Able to be reproduced and extended by other scholars	1) Is it public? Yes 2) Is it available for peer review? Yes, some blogs have pre-publication peer review, others have comments enabled to allow for post-publication peer review) 3) Able to be reproduced and extended by other scholars? Yes, since it is available for review and extendibility since it is openly published on the internet.	1) Is it public? Yes 2) Is it available for peer review? Yes, listeners can leave comments on most podcast hosting sites. 3) Able to be reproduced and extended by other scholars? Yes, since it is available for review and extendibility since it is openly published on the internet.	1) Is it public? Yes 2) Is it available for peer review? Yes, tweetworials can be found by searching Twitter. 3) Able to be reproduced and extended by other scholars? Yes, since it is available for review and extendibility since it is openly published on the internet.
What type of Boyer’s scholarship is this?	Scholarship of teaching	Scholarship of integration (merging of engineering and medicine)	Scholarship of application (helping others to determine if evidence might be applied in their context)
