# Supplementary material for: Consensus Guidelines for Digital Scholarship in Academic Promotion
Source: West J Emerg Med. 2020 Jul 8;21(4):883–91. doi: 10.5811/westjem.2020.4.46441 (PMC7390542; doi:10.5811/westjem.2020.4.46441)
Supplement: Supplementary file 1 [file wjem-21-883-s001.docx]

**Appendix A.** Invited and Actual Attendees of CORD Social Media and Digital Scholarship Committee Consensus Conference on Digital Scholarship in Academic Promotion; April 1, 2019.

Invited to participate:

Felix Ankel

Jennifer Beck-Esmay

Michael Bond+

Emily Brumfield

Daniel Cabrera*

Teresa M. Chan

Rob Cooney

Michael Gisondi

Michael Gottlieb

Abbas Husain

Jay Khadpe

Michelle Lin+

Jess Mason*

Dimitrios Papanagou

Jennifer Repanshek

Zachary Repanshek

Jeffery Riddell

Manny Singh

N. Seth Trueger

Fareen Zaver

*Unable to attend conference but participated significantly in planning and/or post-conference discussion.

+Unable to participate.

Participants were chosen based on their expertise in digital scholarship, their contribution to research and scholarship in the field of digital media, and/or knowledge and participation in promotion and tenure committees. Specific criteria for selection of each participant is listed below:

Felix Ankel is Associate Dean, University of Minnesota Medical School and has been involved extensively in research and education in the realm of digital scholarship.

Jennifer Beck-Esmay is the Associate Editor of R.E.B.E.L EM and Co-Host of the CoreEM Podcast.

Michael Bond is the liaison to the CORD (Council of Residency Directors) Board of Directors from the CORD Social Media Committee.

Emily Brumfield is the Immediate Past Chair of the CORD Social Media Committee.

Daniel Cabrera is a leader in the topic of digital scholarship, including Editor-in-chief for the Mayo Clinic EM Blog, co-director of Mayo Hootsuite Healthcare in Social Media course, and has published multiple pieces of scholarship on the topic.

Teresa M. Chan has been involved extensively in research and education in the realm of digital scholarship including, among others, ALiEM.com, BoringEM.org, EMsimCases.com, ICEnet, MedEdLIFE research collaborative and the METRIQ Study Group.

Rob Cooney is Director Of Faculty Development at Geisinger and Chief Strategy Officer, 2018-20 ALiEM Chief Resident Incubator.

Michael Gisondi is Vice Chair of Education in the Department of Emergency Medicine at Stanford University and has been involved extensively in research and education in the realm of digital scholarship.

Michael Gottlieb is Chief Academic Officer for the Academic Life in Emergency Medicine (ALiEM) Faculty Incubator and Social Media Editor for Academic Emergency Medicine, and representative from CORD iMedEd Track.

Abbas Husain is Chair of the CORD Social Media Committee.

Jay Khadpeis is Past Chair of the CORD Social Media Committee.

Michelle Lin is the creator of Academic Life in Emergency Medicine

Jess Mason is Deputy Editor, EMRAP, Managing Editor of Continuous Core Content (C3), EMRAP-HD, EMRAP Live.

Dimitrios Papanagou is Associate Dean for Faculty Development at Sidney Kimmel Medical College, and Vice Chair of Education, Department of Emergency Medicine.

Jennifer Repanshek is Director of Digital Education and Social Media for the Temple Emergency Medicine Residency at the Lewis Katz School of Medicine/

Zachary Repanshek is a member of the CORD Social Media Committee and Chair of the American Academy of Emergency Medicine Social Media Committee.

Jeffery Riddell is Co-Director, Medical Education Fellowship, USC and an award-winning clinical teacher and researcher in digital innovation.

Manny Singh is Chair Elect of the CORD Social Media Committee, as well as emDOCs.net Co-Founder and Associate Editor-in-Chief, and Assistant Social Media Editor for Academic Emergency Medicine

N. Seth Trueger is Digital Media Editor, JAMA Network Open and has been involved extensively in research and education in the realm of digital scholarship.

Fareen Zaver is Lead Editor/Co-Founder of ALiEM Approved Instructional Resources - Professional (AIR-Pro) and representative from CORD iMedEd Track.
